# Autophagy Genes and Otitis Media Outcomes

**DOI:** 10.3390/clinpract14010023

**Published:** 2024-02-06

**Authors:** Yong Jun Kim, Hwa Sung Rim, Jeong Hee Kim, Sung Soo Kim, Joon Hyung Yeo, Seung Geun Yeo

**Affiliations:** 1 Department of Pathology, College of Medicine, Kyung Hee University, Seoul 02447, Republic of Korea; yjkim1@khu.ac.kr (Y.J.K.); jeonghee3698@naver.com (J.H.K.); 2 Department of Otorhinolaryngology-Head and Neck Surgery, College of Medicine, Kyung Hee University Medical Center, Seoul 02447, Republic of Korea; marslover@naver.com; 3 Department of Biochemistry and Molecular Biology, College of Medicine, Kyung Hee University, Seoul 02447, Republic of Korea; sgskim@khu.ac.kr; 4 Public Health Center, Danyang-gun, Seoul 27010, Republic of Korea; joonhyungyeo@gmail.com

**Keywords:** otitis media, effusion, granulation tissue, cholesteatoma, autophagy, autophagy-related genes

## Abstract

Otitis media (OM) is a common cause of hearing loss in children that requires corrective surgery. Various studies have investigated the pathomechanisms and treatment of OM. Autophagy, an essential cellular recycling and elimination mechanism implicated in various diseases, is known to play an important role in the pathogenesis of OM. Here, we conducted a literature review on autophagy in OM, highlighting the relationship between expression patterns of autophagy-related factors and pathophysiological and clinical aspects of OM. We summarized the existing research results on the expression of autophagy-related factors in acute OM (AOM), OM with effusion (OME), chronic OM (COM) with cholesteatoma, and COM without cholesteatoma (CholeOM) in animals and humans. Autophagy-related factors are expressed in the middle ear mucosa or fluid of AOM, effusion of OME, granulation tissue of COM, and cholesteatoma of CholeOM. Among ATGs and other autophagy-related factors, the most extensively studied in relation to the pathogenesis of OM are mTOR, LC3II/I, PI3K, Beclin-1, FLIP, Akt, and Rubicon. Expression of autophagy-related factors is associated with AOM, OME, COM, and CholeOM. Inadequate expression of these factors or a decrease/increase in autophagy responses can result in OM, underscoring the critical role of ATGs and related factors in the pathogenesis of OM.

## 1. Introduction

### 1.1. Otitis Media

**Figure 1:**
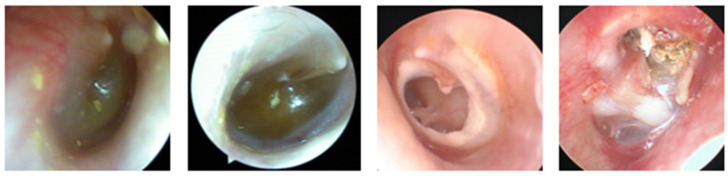
Tympanic membrane findings of acute otitis media (AOM), OM with effusion (OME), chronic OM (COM) and cholesteatomatous OM (CholeOM).

Otitis media is divided into acute, subacute, and chronic depending on its duration after onset. Otitis media is considered acute if it lasts 3 weeks after onset, subacute if it last 3 weeks to 3 months, and chronic if it persists for more than 3 months. If inflammatory fluid accumulates in the middle ear cavity without a perforation in the eardrum, it is called otitis media with effusion. In cases where there is a perforation in the eardrum and exudation of purulent discharge, it is referred to as suppurative otitis media. Chronic otitis media, in turn, can be classified as chronic otitis media with cholesteatoma or chronic otitis media without cholesteatoma (Figure 1). Otitis media is associated with histological changes caused by inflammatory reactions in the eardrum and middle ear cavity [1].

Acute otitis media and otitis media with effusion can cause structural changes in the tympanic membrane, demonstrated by histological analysis of inner and outer fibrous layers of the lamina propria. These changes affect the elasticity of the eardrum, creating a condition in which collapse or perforation of the eardrum is possible. Acute inflammation of the middle ear causes pathological transformation and hyperplasia of the middle ear mucosa. In the case of acute stimulation, both hyperplasia of the middle ear mucosa and influx of various inflammatory cells into the mucosa are largely reversible, such that the mucosa returns to its normal appearance through a de-differentiation process after the stimulation associated with otitis media disappears. However, when pathological conditions, such as middle ear mucosal hyperplasia, middle ear effusion due to hyperproliferative reactions, mineralized atelectasis, adhesions, tympanic sclerosis, and middle ear cholesteatoma, occur repeatedly and become chronic, they result in irreversible structural changes in the middle ear cavity. Pathological conditions, such as middle ear mucosal hyperplasia, middle ear effusion due to hyperproliferative reactions, mineralized atelectasis, adhesions, tympanic sclerosis, and middle ear inflammation, were found to occur repeatedly and become chronic, resulting in irreversible structural changes in the middle ear cavity. Although acute otitis media is usually resolved without sequelae, some patients may experience the recurrence or persistence of inflammation, progressing to recurrent otitis media, otitis media with effusion, or a form of chronic otitis media [2,3].

In chronic otitis media, various histopathological changes occur in the middle ear and mastoid. Some of these changes appear as a direct result of infection, whereas others occur as a result of the host’s immune response. Chronic otitis media without cholesteatoma causes changes in the mucosa, submucosa, and surrounding bone tissue as a result of a persistent inflammatory response in the middle ear cavity or mastoid. Important pathological findings include the formation of granulation tissue, bone changes, tympanosclerosis, cholesterol granuloma, and fibrosis. These lesions vary depending on whether chronic otitis media is active or inactive, and are often found even in the absence of tympanic membrane perforation. When chronic otitis media is active, a considerable amount of granulation tissue, rich in cellular components or vascular tissue, is found [4,5]. Bone changes appear in the form of fixation or erosion of the ossicles. The frequency of ossicular lesions is higher in patients with the active form of chronic otitis media accompanied by otorrhea than in patients with the inactive form of chronic otitis media without otorrhea. Destruction of the ossicles occurs most often at the incudostapedial joint. Among ossicles, the rank order of involvement is the incus, stapes, and malleus; however, the area of the ossicles that shows bone destruction may vary depending on the form or type of chronic otitis media [6].

Cholesteatoma is a condition in which keratinized squamous epithelium invades the middle ear cavity, composed of the mucosa. The resulting accumulation of keratin, in association with desquamation of the epidermis, causes destruction of surrounding bone tissue. This destruction can be readily observed, especially in cholesteatomatous otitis media. The process underlying bone destruction involves the pressure effect of the cholesteatoma itself as well as the action of various enzymes, including collagenase, acid phosphatase, and acid protease secreted from the granulation tissue caused by the inflammatory reaction; various cytokines secreted by inflammatory cells are also known to act. These various mechanisms are considered to interact with each other to activate local osteoclasts and cause bone destruction [7,8]. Several hypotheses regarding the mechanism underlying the occurrence of cholesteatoma have been proposed. In the case of congenital cholesteatoma, these include the defective tympanic annulus theory, the epidermoid formation theory, the epithelial rest theory, and the squamous metaplasia theory. In the case of acquired cholesteatoma, four theories have been proposed: the invagination theory, the epithelial invasion theory, the basal cell hyperplasia theory, and the squamous metaplasia theory. Cholesteatoma, or granulation tissue, is known to be an important clinical cause of hearing loss, vertigo, facial nerve paralysis, and intracranial complications by causing bone destruction in chronic otitis media [4,5].

Otitis media, which incurs significant social and economic costs, is one of the most common diseases in children. Recurrent or chronic otitis media with effusion causes hearing loss, which can cause speech development disorders, language acquisition delays, attentional distraction, and behavioral abnormalities in children. Considerable research effort has been devoted to understanding the roles of otitis media-causing factors, inflammatory responses, inflammatory mediators, innate immune responses, and adaptive immune responses in otitis media, and rapid progress has been made in identifying the pathogenesis and pathophysiology of this pathology. However, despite this effort, various aspects of otitis media remain incompletely understood, impacting the efficacy of treatments.

### 1.2. Autophagy

Autophagy, a protein degradation system, is a catabolic process that acts as a stress-response or is a natural way to destroy and recycle unnecessary or damaged components within cells. The word “autophagy” is from the Greek for “self-eating”, with this process being evolutionarily conserved in all eukaryotic cells. The general purpose of autophagy is to maintain cellular homeostasis and quality control by removing damaged or unnecessary cellular components, including organelles and proteins, through a controlled degradation process. It has been consistently recognized that autophagy plays an important role in regulating a variety of human physiological and pathological processes, including cell growth, differentiation, metabolic regulation, cancer, brain disease and immunity. Given its diverse roles, autophagy is unsurprisingly the subject of ongoing studies by numerous researchers. Autophagy can be divided into three types—macroautophagy, microautophagy, and chaperone-mediated autophagy—depending on its operating mechanism and function. These three types of autophagy coexist within cells and each is induced differently. Broadly speaking, the term autophagy generally refers to macroautophagy. In an environment where intracellular nutrients are lacking, this process triggers the cell to surround its own unnecessary proteins and aged organelles with a membrane vacuole (autophagosome) composed of a lipid bilayer, which then combines with lysosomes to induce intracellular decomposition of the enclosed substances. In this scenario, a nutrient resupply system plays an important role in maintaining cellular homeostasis. Autophagy is classified in various ways depending on the type of cargo being decomposed. Mitophagy protects cells from damage by selectively removing damaged mitochondria; autophagy can also be classified into ribophagy, reticulophagy, and pexophagy depending on whether the process decomposes ribosomes, endoplasmic reticulum, or peroxisomes [9,10]. Autophagy has received considerable attention for its role in disease, a role that has been revealed through studies of various animal models of disease. However, much about the specific mechanisms and functions of the molecules involved remains unknown.

**Figure 2:**
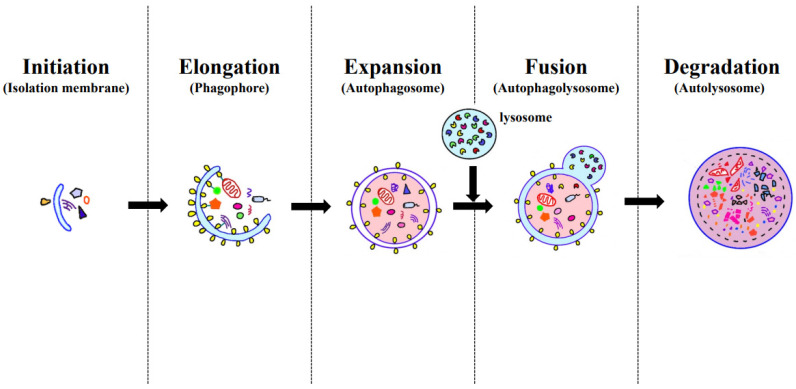
The autophagy pathway.

Autophagy is a highly regulated cellular process that involves the degradation and recycling of cellular components and organelles to maintain cellular homeostasis and respond to various stress conditions. It is a dynamic process that can be broken down into several stages: initiation, elongation (phagophore formation), expansion (autophagosome formation), fusion (autophagolysosome formation), and degradation (autolysosome formation) (Figure 2). Initiation: Autophagy is initiated in response to various cellular signals and stressors, such as nutrient deprivation, oxidative stress, or the presence of damaged organelles or protein aggregates. The key regulatory protein involved in initiation is the mammalian target of rapamycin (mTOR), which is inhibited when cells sense stress signals. Inhibition of mTOR leads to the activation of the unc-51-like autophagy activating kinase 1 (ULK1) complex, which is essential for the initiation of autophagy. The ULK1 complex phosphorylates various downstream targets, including Beclin-1, to promote the nucleation of the autophagic vesicle. Elongation (phagophore formation): The elongation stage involves the formation of a double-membraned structure called the phagophore, also known as the isolation membrane. Phagophore formation starts at specific sites called omegasomes, which are rich in phosphatidylinositol 3-phosphate (PI3P). Autophagy-related protein 6 (ATG6/Beclin-1) and ATG14 play important roles in promoting the formation of PI3P-rich membranes. The phagophore expands by the addition of lipid molecules from various sources, such as the endoplasmic reticulum (ER) or lipids synthesized de novo. Expansion (autophagosome formation): The phagophore continues to elongate and eventually engulfs the cellular cargo that needs to be degraded, such as damaged organelles or protein aggregates. ATG5-ATG12 and ATG8 (LC3) conjugation systems are crucial for the expansion and sealing of the phagophore to form the autophagosome. The LC3 protein, in its lipidated form (LC3-II), is incorporated into the autophagosomal membrane and serves as a marker for autophagosomes. Fusion (Autophagolysosome Formation): Once the autophagosome is formed, it needs to fuse with lysosomes to become an autophagolysosome, where degradation can occur. The autophagosome fuses with lysosomes, which contain the hydrolytic enzymes and acidic conditions necessary for degradation. Fusion is mediated by SNARE proteins and regulatory factors, ensuring that the contents of the autophagosome are delivered to the lysosome. Degradation (Autolysosome): In the autolysosome, the sequestered cargo is exposed to lysosomal enzymes, including proteases, nucleases, and lipases. These enzymes break down the cargo into its constituent molecules, such as amino acids, nucleotides, and fatty acids, which can then be recycled or used by the cell to maintain its functions. The breakdown products are transported back to the cytoplasm through lysosomal membrane transporters, where they can be reused for cellular processes. In contrast to macroautophagy, microautophagy is a process in which single-membrane vesicles containing cargo are directly engulfed and decomposed by the lysosomal membrane [11,12]. The third type of autophagy, chaperone-mediated autophagy, is responsible for the degradation of approximately 30% of soluble proteins in the liver and kidneys. Macroautophagy and microautophagy remove intracellular substances through selective and non-selective mechanisms, whereas chaperone-mediated autophagy is a process activated by nutritional deficiency or various toxic compounds that selectively degrade cytoplasmic proteins [13,14].

The autophagy mechanism for decomposing cargo in cytoplasmic lysosomes, which is important for maintaining homeostasis in cells, tissues and organisms, is controlled by evolutionarily conserved autophagy-related genes (ATGs). Genetic mutations that regulate autophagy have clear causal relationships with diseases such as neurodegeneration, inflammatory diseases, and cancer. Since autophagy selectively targets abnormal cellular components, intracellular microorganisms and pathogenic proteins, any abnormality in this process can cause disease. In addition to their specific functions in autophagy, ATGs play various important physiological roles in membrane transport and signaling pathways [15,16,17,18,19]. The primary identified function of ATGs is to regulate the formation of double-membrane structures that transport intracellular materials to lysosomes for degradation. This process is conserved in all eukaryotic organisms, occurs natively in almost all cell types, and is augmented by a variety of intracellular and extracellular signals. It also is essential for cellular homeostasis, quality control of cellular proteins and tissues, and adaptation to environmental stresses [11,20,21].

How autophagy is directly involved in maintaining human health or developing disease is still unclear. Various studies are underway to determine whether autophagy has a positive or negative effect on human diseases, but the mechanisms are more complex than expected [22]. As part of this research, attempts are being made to treat or prevent diseases by controlling the expression of autophagy-related factors. Recent studies have shown that failure to properly express and activate autophagy is associated with the development of various diseases, and the absence of proper induction of autophagy is known to play an important role in the occurrence and pathogenesis of otitis media. Accordingly, to determine how autophagy affects the pathophysiology and clinical aspects of otitis media, we conducted a review of the literature on patterns of ATG and related factor expression in otitis media.

Our search encompassed various literature databases. From 2018 to 2023, we identified nine studies based on search terms from four electronic databases: (a) SCOPUS, (b) PubMed, (c) EMBASE and (d) the Cochrane Library. Search terms included “autophagy” and “otitis media”. Off topic studies (i.e., other than otitis media), reviews articles, and studies in languages other than English were excluded. Studies were included if they (1) were prospective or retrospective investigations of autophagy and otitis media; (2) included in vitro studies on autophagy and otitis media; and (3) included human patients and/or animal studies on autophagy and otitis media. Seven studies met the criteria for otitis media and were included in this review: two studies involving patients with acute OM (AOM), one involving patients with OM with effusion (OME), one involving patients with chronic OM (COM), one involving patients with cholesteatomatous OM (CholeOM) and two involving patients with COM and CholeOM.

## 2. Autophagy in Otitis Media

### 2.1. Acute Otitis Media (AOM)

In a recent study [23], a mouse model of OM was created using Tlr2tm1Kir (TLR2−/−) mice, lacking the Tlr2 (Toll-like receptor 2) gene, inoculated in the middle ear (ME) with streptococcal pneumonia peptidoglycan polysaccharide (PGPS). TLR2−/− mice and wild-type (WT) mice (both 6–8 weeks old) were divided into four groups and injected intraperitoneally with normal saline (NS), PGPS (streptococcal peptidoglycan-polysaccharide), NS + dimethyl sulfoxide (DMSO), or PGPS + rapamycin (RPM), the latter of which is a widely used mammalian/mechanistic target of rapamycin complex 1 (mTORC1) inhibitor and autophagy inducer. On the third day following injection, eardrums of the experimental mice were examined using an otoscopic digital imaging system and subsequently sacrificed. Bullae tissues from the inner ear were then collected and examined by histological analysis, immunohistochemistry, immunofluorescence, and TUNEL (terminal deoxynucleotidyl transferase-mediated dUTP-biotin nick-end labeling) assay. Protein expression levels of p-S6, Raptor, and mTOR were decreased in TLR2−/− mice after PGPS injection. Additionally, the autophagosome proteins, i.e., microtubule-associated protein 1A/1B-light chain 3 (LC3)-II, beclin-1 and ATG7, and autophagosome substrate protein p62, accumulated at higher levels in mice with OM than in OM-negative mice. Expression of the lysosomal-associated proteins, i.e., LAMP1, cathepsin B and cathepsin D, was increased in OM mice compared with OM-negative mice. After RPM treatment, the accumulation of LC3-II, Beclin-1, and ATG7 decreased, and the expression of Rab7 and syntaxin 17 significantly increased. These results suggest that PGPS-induced OM is associated with autophagic damage, and that RPM improves OM, at least in part, by alleviating this damage. They further suggest that modulating autophagic activity by RPM could be an effective treatment strategy for OM in animals [23].

Similar results were obtained in a previously published animal study employing a mouse model of AOM created by transbullar injection of Streptococcus pneumonia (S.pn.) [24]. In this study, the production of autophagy in response to S.pn was evaluated in vivo and in vitro by measuring the conversion of the autophagy protein LC3-I to LC3-II, which serves as an indicator of autophagy activation and autophagosome formation. Thirty minutes after incubation with S.pn, both LC3-II expression and the number of LC3 puncta per cell were significantly increased. Treatment of neutrophils with the autophagy inhibitor, 3-MA (3-methyladenine), significantly inhibited the conversion of LC3-I to LC3-II and abolished the generation of LC3 puncta. In this mouse model of AOM, the expression of LC3-II was significantly increased on days 1 and 3 after culture with S.pn., suggesting that autophagy is a major contributor to the pathogenesis of S.pn-induced OM in vivo and in vitro (Table 1).

### 2.2. Otitis Media with Effusion (OME)

Among the many studies that have sought to uncover the etiology of OME, recent work has increasingly focused on the role of autophagy. In this context, we have investigated whether the expression of autophagy-associated mRNAs in effusion fluid of adults and children differs depending on the clinical pattern [25]. In this study, fluid retained in the middle ear was collected from 61 children and 36 adults with OME during surgery, and the expression of autophagy-associated mRNAs was analyzed by quantitative real-time RT-PCR and related to the results of bacterial culture tests, number of ventilating tube insertions, and middle ear fluid characteristics. The autophagy-related factors selected for analysis included mTOR, which is involved in induction and initiation, beclin-1, which is involved in nucleation, FLIP (FADD-like IL-1β-converting enzyme inhibitory protein), which is involved in vesicle elongation and antagonizes ATG3, and Rubicon, which inhibits maturation. Notably, among these autophagy-related mRNAs, beclin-1 mRNA was significantly decreased in children compared with adults, where beclin-1 mRNA was significantly increased in reservoir fluid without bacteria, regardless of the number of surgeries or the nature of the reservoir fluid. Thus, although autophagy-related RNAs were found to be expressed in the effusion fluid retained in the middle ear in both adults and children with OME and was involved in the pathogenesis of OME, why only beclin-1 mRNA was increased in the fluid of adults and decreased in children remains unexplained (Table 1).

### 2.3. Chronic Otitis Media without Cholesteatoma (COM)

Turning to the role of autophagy in COM, a recent study sought to compare and analyze differences in the expression of autophagy-related mRNAs in relation to various clinical presentations in patients undergoing surgery for COM without cholesteatoma [26]. In this study, granulation tissue from the middle ear was collected from 47 patients during surgery and analyzed for autophagy-related mRNA expression using quantitative RT-PCR. The specific objectives were to determine whether expression of autophagy-related mRNAs differed depending on the presence or absence of bacteria, otorrhea, conductive hearing loss, or sensorineural hearing loss. All inflamed COM tissues showed expression of autophagy mRNAs, with LC3II mRNA displaying the highest expression, followed by beclin-1, PI3K, Rubicon, and mTOR mRNAs. Bacterial cultures performed on tissue from the middle ear cavity revealed a 46.8% positivity rate for Pseudomonas aeruginosa, the most common strain detected, with lesser positivity rates for (in order) methicillin-resistant Staphylococcus aureus (MRSA), coagulase-negative S. aureus, S. aureus and *Achromobacter xylosoxidans*. The expression of beclin-1 mRNA was found to be significantly lower in the bacteria-positive group compared with the bacteria-negative group (*p* < 0.05). In contrast, there were no significant associations between the expression of autophagy-related mRNAs and the presence or absence of otorrhea or type of hearing loss. These results confirm that all autophagy-related mRNAs tested were expressed in COM, implicating autophagy in the pathogenesis of OM. However, there were several notable limitations of this study. First, there was no normal control group because obtaining normal middle ear mucosa was impractical. Second, only mRNA levels were tested without consideration of protein levels. Third, even some patients in the group classified as otorrhea negative owing to the absence of otorrhea in the external auditory canal showed inflammatory reactions and otorrhea in the middle ear and mastoid process at the time of surgery. Fourth, expression of autophagy-related mRNAs might have been affected by presurgical use of antibiotics for 1 week. Finally, it was not possible to determine which changes in the lesions and clinical features of OM depended on the activation and inactivation of autophagy-related factors (Table 1).

### 2.4. Chronic Otitis Media with Cholesteatoma (CholeOM)

Although autophagy is associated with several diseases, whether it also plays a role in cholesteatoma is largely unknown. In a recent study addressing this question [27], paired retroauricular skin (control subject) and cholesteatoma (CholeOM patient) samples from 15 patients, collected during surgery, were analyzed for LC3, phosphorylated Akt (p-Akt), and phosphorylated mTor (p-mTOR) by Western blot analysis. Expression of LC3, p-Akt, and p-mTOR was also analyzed in post-auricular skin and cholesteatoma samples from 10 patients using immunohistochemistry. Expression of the commonly used autophagy indicator, LC3, was significantly decreased in cholesteatoma in at least 10 of 15 pairs of samples. Because the Akt/mTOR pathway is an upstream negative regulator of autophagy, p-Akt/p-mTOR expression was investigated in 15 pairs of samples for possible upregulation in cholesteatomas, but no clear increasing trend in the expression of p-Akt or p-mTOR was detected, possibly indicating that the Akt/mTOR pathway is not involved in the observed down-regulation of autophagy. Immunohistochemical analyses demonstrated that 9 of 10 postauricular skin tissues were positive for LC3 staining, but only 3 of 10 cholesteatomas showed positive LC3 staining, a difference that was significant. These findings suggest an inverse correlation between LC3 expression and cholesteatoma, pointing to an autophagy deficiency in cholesteatoma. Notably, this suppressed autophagy in cholesteatoma tissue appears to be linked to LC3 rather than the Akt/mTOR signaling pathway (Table 1).

### 2.5. Chronic Otitis Media without Cholesteatoma (COM) versus Chronic Otitis Media with Cholesteatoma (CholeOM)

Available evidence suggests that the role of autophagy in COM differs depending on whether it is accompanied by cholesteatoma. A recent study took aim at this question [28], collecting inflammatory tissue, classified into granulation tissue and cholesteatoma according to tissue biopsy results, from 47 patients undergoing surgery for chronic otitis media. Differences in expression of autophagy-related mRNAs between non-cholesteatomatous COM and CholeOM were measured using quantitative RT-PCR and related to the presence of bacteria or otorrhea and degree of hearing loss. In this study, it was not possible to investigate all autophagy-related factors and their adapter molecule mRNAs. Instead, mTOR (involved in the initiation process), PI3K and beclin-1 (involved in vesicle nucleation), LC3 II (involved in vesicle elongation), and Rubicon (involved in the fusion and degradation process) were selected for study. The expression of PI13K mRNA was higher in the COM group than in the CholeOM group, and the expression of beclin-1 mRNA was higher in the CholeOM group than in the COM group (*p* < 0.05). In the group testing positive for bacteria, P13KC3 mRNA expression was higher in COM samples than in CholeOM samples. Conversely, in the bacteria-negative group, the expression of beclin-1 mRNA was higher in samples from CholeOM patients than in those from COM patients (*p* < 0.05). In cases presenting with otorrhea and hearing loss greater than 40 dB HL, P13KC3 mRNA expression was higher in the COM group than in the CholeOM group (*p* < 0.05). Collectively, the findings of this study demonstrated that, among various autophagy-related factors, PI3KC3 mRNA was significantly increased in the COM group, and beclin-1 mRNA was significantly increased in the CholeOM group, indicating that the differential expression of autophagy-related factors in COM depends to a large extent on whether cholesteatoma is present.

However, another study comparing the role of autophagy in COM and CholeOM patients [29] reached different conclusions. In this study, cholesteatoma epithelium and normal external auditory canal (EAC) epithelium were collected from 15 patients with CholeOM; the marginal epithelium of tympanic membrane perforation was also collected from 15 patients with COM. Immunohistochemistry, used to detect the expression of LC3 in cholesteatoma and EAC epithelium, showed positive staining for LC3A and LC3B in both cholesteatoma epithelium and EAC epithelium, with stronger staining in the cholesteatoma epithelium than in normal EAC epithelium. These experiments further showed that LC3A/B was mainly expressed in the keratinizing squamous epithelium, with stronger staining in the basal layer. Semi-quantitative analysis of protein expression by Western blotting showed that LC3A-II/I and LC3B-II/I ratios were significantly higher in the cholesteatoma epithelium than in EAC epithelium (*p* = 0.001). Additionally, beclin-1 expression levels were significantly higher in the cholesteatoma epithelium than in EAC epithelium (*p* = 0.001). To identify the autophagy signaling pathway, the authors of this study also investigated p-PI3K, p-AKT, and AKT expression using Western blotting, finding significantly higher p-PI3K/PI3K (*p* = 0.001) and p-AKT/AKT (*p* = 0.001) ratios in the cholesteatoma epithelium than in EAC epithelium. These results show that autophagy may play an important role in the pathogenesis of cholesteatoma (Table 1).

## 3. Conclusions

Otitis media is one of the most common diseases in children and is a disease that incurs significant social and economic costs. Recurrent or chronic otitis media with effusion causes hearing loss, which can cause speech development disorders, language acquisition delays, attentional distraction, and behavioral abnormalities in children. To date, various studies have been conducted to understand the roles of causative factors, inflammatory responses, inflammatory mediators, innate immune responses, and adaptive immune responses in otitis media, and rapid progress has been made in identifying the pathogenesis and pathophysiology of this pathology. However, these efforts have fallen short of completely resolving problems relating to immune system involvement in otitis media. Accordingly, considerable current research is focused on understanding the immunological mechanisms in the middle ear cavity and how these insights might be used to treat otitis media. Autophagy also appears to be an important player in the occurrence and pathogenesis of otitis media. This conclusion is reinforced by our literature review, which supports the involvement of mTOR, LC3II/I, PI3K, beclin-1, FLIP, Akt, and Rubicon in the pathogenesis of otitis media. Collectively, these studies implicate inappropriate expression of these autophagy-related factors, or a decrease or increase in autophagy activation, in the pathogenesis of acute otitis media, otitis media with effusion, chronic otitis media, and cholesteatomatous otitis media. It is hoped that future studies will clearly establish the pathophysiology of autophagy-related factors in otitis media, leading to the treatment of otitis media.

## Figures and Tables

**Table 1:** Expression of autophagy-related substances in otitis media.

Author /Year [Reference]	Species	Sample	Type of OM	Detection Method	Target Substance(s) Associated with Autophagy	Results Conclusion
Xie D, et al., 2022 [23]	TLR2−/− mice and WT mice	Middle ear	AOM	Histological analysis, immunohistochemistry, immunofluorescence, TUNEL assays	mTOR, LC3-II, beclin-1, ATG7	Protein expression levels of p-S6, Raptor and mTOR are decreased in TLR2−/− mice after injection of PGPS. Both the autophagosome proteins LC3-II, beclin-1 and ATG7, and autophagy substrate protein p62 accumulated at higher levels in mice with OM than in OM-negative mice. Impaired autophagy is involved in PGPS-induced OM, which is improved by RPM, at least in part, by relieving autophagy impairment.
Dong Y, et al., 2017 [24]	C57BL/6 mice	Middle ear	AOM	NETs killing assay, confocal microscopy NETs capture effect assay	LC3-I LC3-II	LC3-II expression is significantly increased 30 min after incubation with S.pn. In addition, the numbers of LC3 puncta per cell significantly increase following S.pn infection. In the course of AOM, TLR4 controls NET formation against S.pn through regulation of autophagy.
Kim SH, et al., 2018 [25]	Humans	Effusion	OME	Quantitative PCR, bacterial culture	mTOR, beclin-1, FLIP, Rubicon	Beclin-1 mRNA levels were significantly lower in pediatric than adult patients, regardless of the frequency of surgery or fluid characteristics (*p* < 0.05). Autophagy-associated mRNAs were expressed in effusion fluids of both pediatric and adult patients with OME. However, the level of beclin-1 mRNA was significantly lower in the effusion fluid of pediatric than adult patients.
Jung J, et al., 2020 [26]	Humans	Inflammatory tissues	COM	Quantitative PCR, bacterial culture	mTOR, P13KC3, LC3 II, beclin-1, Rubicon	Autophagy-related mRNAs were detected in all inflammatory tissues of COM patients. LC3-II mRNA expression was highest, followed by expression of beclin-1, P13KC3, Rubicon, and mTOR mRNAs. Beclin-1 mRNA levels were significantly lower in culture-positive than culture-negative patients. Autophagy is involved in the pathogenesis of COM. The finding that the expression of autophagy markers, especially beclin-1, was lower in culture-positive than culture-negative patients suggests that these markers are closely associated with the clinical features of COM.
Ho KY, et al., 2020 [27]	Humans	Retroauricular skin and cholesteatoma tissue	CholeOM	Immunoblotting, immunohistochemistry, MTT assays	LC3, Akt, mTOR	Expression of LC3, p-Akt, and p-mTOR in fresh retroauricular skin and cholesteatoma tissue samples was analyzed by immunoblotting and immunohistochemistry. Autophagy is significantly suppressed in cholesteatoma tissues and may not involve the Akt/mTOR signaling pathway.
Jung J, et al., 2020 [28]	Humans	Inflammatory tissues	COM CholeOM	Quantitative PCR, bacterial culture	mTOR, PI3KC3, LC3 II, beclin-1, Rubicon	PI3K mRNA expression was lower, whereas Beclin-1 mRNA expression was higher (0.089 ± 0.107 vs. 0.176 ± 0.163; *p* = 0.034) in the CholeOM group. PI3K mRNA expression was lower in the CholeOM group than in COM subgroups presenting with bacterial infection, otorrhea, and hearing loss > 40 dB. Different autophagy proteins play important roles in chronic OM depending on the presence or absence of cholesteatoma.
Li Q, et al., 2019 [29]	Humans	Cholesteatoma epithelium and normal external auditory canal epithelium	COM CholeOM	Immunohistochemistry, Western blotting	LC3, beclin-1, PI3K/AKT pathway	LC3 immunostaining was stronger in cholesteatoma epithelium than in normal EAC epithelium. Western blotting showed significantly higher LC3-II/I ratios and Beclin-1 expression in cholesteatoma epithelium compared with EAC epithelium or COM epithelium, and significantly higher p-PI3K/PI3K and p-AKT/AKT ratios in cholesteatoma epithelium compared with EAC epithelium. Enhanced autophagy might play a role in the pathogenesis of acquired cholesteatoma. PI3Ks might have different regulatory functions in autophagy in the cholesteatoma epithelium.

AMPK, activated protein kinase; Akt, protein kinase B; CholeOM, cholesteatoma; COM, chronic otitis media; FLIP, FADD-like IL-1β-converting enzyme inhibitory protein; LC 3; microtubule-associated protein-1 light chain 3 protein; 3-MA; 3-methyladenine; mTOR, mammalian target of the rapamycin; NETs, neutrophil extracellular traps; OM, otitis media; OME, otitis media with effusion; PI3K, phosphatidylinositol 3-kinase; RT-PCR, reverse transcription-polymerase chain reaction; qRT-PCR, quantitative RT-PCR; S.pn, *Streptococcus pneumoniae*; TDT, Terminal Deoxynucleotidyl Transferase.

## Data Availability

Not applicable.
